# Roles of Direct Photoreception and the Internal Circadian Oscillator in the Regulation of Melatonin Secretion in the Pineal Organ of the Domestic Turkey: A Novel In Vitro Clock and Calendar Model

**DOI:** 10.3390/ijms20164022

**Published:** 2019-08-17

**Authors:** Magdalena Prusik, Bogdan Lewczuk

**Affiliations:** Department of Histology and Embryology, Faculty of Veterinary Medicine, University of Warmia and Mazury in Olsztyn, Oczapowskiego 13, Olsztyn 10-719, Poland

**Keywords:** pineal organ, melatonin, biological rhythm, turkey, birds

## Abstract

The regulation of melatonin secretion in the avian pineal organ is highly complex and shows prominent interspecies differences. The aim of this study was to determine the roles of direct photoreception and the internal oscillator in the regulation of melatonin secretion in the pineal organ of the domestic turkey. The pineal organs were collected from 12-, 13- and 14-week-old female turkeys reared under a 12 L:12 D cycle with the photophase from 07.00 to 19.00, and were incubated in superfusion culture for 3–6 days. The cultures were subjected to different light conditions including 12 L:12 D cycles with photophases between 07.00 and 19.00, 13.00 and 01.00 or 01.00 and 13.00, a reversed cycle 12 D:12 L, cycles with long (16 L:8 D) and short (8 L:16 D) photophases, and continuous darkness or illumination. The pineal organs were also exposed to light pulses of variable duration during incubation in darkness or to periods of darkness during the photophase. The secretion of melatonin was determined by direct radioimmunoassay. The turkey pineal organs secreted melatonin in a well-entrained diurnal rhythm with a very high amplitude. Direct photoreception as an independently acting mechanism was able to ensure quick and precise adaptation of the melatonin secretion rhythm to changes in light-dark conditions. The pineal organs secreted melatonin in circadian rhythms during incubation in continuous darkness or illumination. The endogenous oscillator of turkey pinealocytes was able to acquire and store information about the light-dark cycle and then to generate the circadian rhythm of melatonin secretion in continuous darkness according to the stored data. The obtained data suggest that the turkey pineal gland is highly autonomous in the generation and regulation of the melatonin secretion rhythm. They also demonstrate that the turkey pineal organ in superfusion culture is a valuable model for chronobiological studies, providing a highly precise clock and calendar. This system has several features which make it an attractive alternative to other avian pineal glands for circadian studies.

## 1. Introduction

Daily and seasonal changes in the environmental photoperiod are reflected in the rhythmicity of many physiological processes and behaviors in vertebrates. Melatonin (MLT) produced by the pineal organ is considered to be the main chronobiotic hormone, which acts as a clock molecule, because of the diurnal rhythm of its concentration in blood [1,2,3,4,5,6,7]. High level of MLT is a chemical signal of night and darkness. Throughout the year, duration of the elevated MLT concentration in the general circulation changes parallel to the seasonal alternations in the night length, which enables MLT to be a calendar molecule [2,6,8,9,10,11]. 

It is well-documented that the environmental light is received directly by avian pinealocytes, which possess own photoreceptive structures and phototransduction molecules [12,13,14,15,16,17]. Among photopigments, pinopsin is characteristic for the pineal organ, while others, including melanopsin, occur both in the pineal organ and the retina [12,13,14]. The environmental light also controls the activity of the avian pineal organ by the indirect pathway via the retina, the retinohypothalamic tract, the suprachiasmatic nucleus (SCN) and the sympathetic fibers [18,19]. The rhythm of MLT secretion, although strongly related to the environmental light-dark conditions, is partially protected against the influence of accidental exposition to light or darkness. The stability of MLT rhythm is ensured by the endogenous oscillators, being the autoregulatory systems of clock genes encoding periods of light and dark phases of the daily cycle [6,9,20,21,22]. The circadian oscillators (also known as chronometers, timekeepers, or clocks) are self-sustained and continue their functions even in the absence of light input from the environment [2,6,7,21,23,24,25]. In most examined birds, the circadian oscillators are located in the pineal gland, SCN, and retina [2,9,15,19]. Three oscillators cooperate with each other in birds and form a centralized system keeping daily time.

The most popular avian model for both in vivo and in vitro studies on the mechanisms regulating MLT secretion is the chicken [10,18,21,22,26,27,28,29,30,31,32,33,34,35,36]. Infrequent studies on other birds have shown that the functional significance of the direct and indirect routes of the effect of light on pineal organ activity as well as the contribution of each clock in the regulation of MLT secretion differ significantly between avian species [2,6,9,15]. These differences are not be surprising because the avian pineal organs show among the greatest variability of morphology observed among vertebrates [37,38,39,40,41,42,43,44,45,46,47,48]. Concerning poultry species, our in vitro studies on the pineal organs of *Anseriformes* birds demonstrated that light acting directly on pinealocytes is not able to ensure the precise entrainment of MLT secretion to a light-dark cycle and that norepinephrine is the main factor controlling pineal organ activity in the duck and the goose [7,25]. The interest in the turkey pineal organ derives from at least two factors. First, the histological organization and ultrastructure of the turkey pineal organ differ greatly from those of the chicken pineal organ, despite the fact that both species belong to the *Galliformes* family [37,38,41,43,45]. The turkey pineal organ retains a tubule-follicular structure up to the age one year and possess highly developed rudimentary-receptor pinealocytes, which lead to the hypothesis that direct photoreception plays a primary role in the regulation of MLT secretion in this organ [45]. Second, the turkey is a poultry species with high economic significance and knowledge about its circadian physiology may be important from a practical point of view [49,50,51]. 

The aim of the present study was to determine the roles of direct photoreception and the intrapineal oscillator in the regulation of MLT production in the turkey pineal organ. The obtained results showed that the turkey pineal organ is a valuable model for chronobiological studies in vitro.

## 2. Results

### 2.1. Experiment I

**Figure 1:**
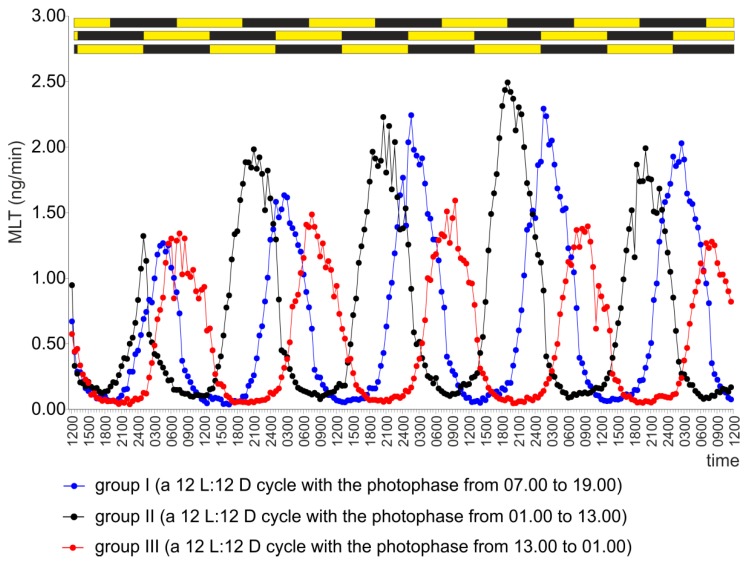
Experiment I. Secretion of MLT (mean) from turkey pineal organs incubated under a 12 L:12 D cycle with the photophase from 07.00 to 19.00 (group I), a 12 L:12 D cycle with the photophase from 01.00 to 13.00 (group II), or a 12 L:12 D cycle with the photophase from 13.00 to 01.00 (group III) during five consecutive days.

Pineal organs incubated under a 12 L:12 D cycle with the light phase from 07.00 to 19.00 (group I), which was the same light-dark cycle used at the time of turkey rearing, secreted MLT in a regular diurnal rhythm throughout the experiment (Figure 1). MLT secretion increased in a step-wise manner after the onset of scotophase, reached the highest level between 01.00 and 04.00, and then slowly decreased (Figure S1). The lowest level of secretion was observed between 12.00 and 16.00. The amplitude of the rhythm was approximately 20-fold during the first day of the experiment and approximately 40-fold during subsequent days of culture (Table 1). 

The secretion of MLT from the pineal organs incubated under a 12 L:12 D cycle with the light phase between 01.00 and 13.00 (group II) was characterized by a forward shift of the rhythm by 5–6 hours compared with group I starting on the second day of the experiment (Figure 1, Table 1).

The pineal organs incubated under a 12 L:12 D cycle with the photophase between 13.00 and 01.00 (group III) exhibited rhythmic changes in MLT secretion delayed by 5–6 hours compared with group I from the first day of the experiment (Figure 1, Table 1). 

**Figure 2:**
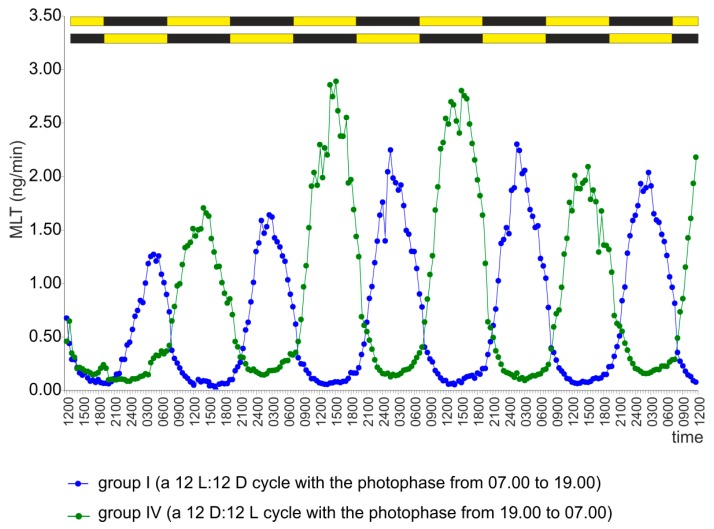
Experiment I. Secretion of MLT (mean) from turkey pineal organs incubated under a 12 L:12 D cycle with the photophase from 07.00 to 19.00 (group I) or a reversed cycle of 12 D:12 L (group IV) during five consecutive days.

In the group of pineal organs incubated under a reversed light-dark cycle 12 D:12 L (group IV), a small increase in MLT secretion was noted at the end of the first scotophase (Figure 2). MLT secretion decreased after exposure of the pineal organs to light; however, it increased again in the second half of the first photophase. From the second day onward, the MLT secretion rhythm was completely reversed compared with the group incubated under a 12 L:12 D cycle with the photophase between 07.00 and 19.00 (Figure 2, Table 1). Maximum secretion was noted between 14.00 and 15.00 and minimum secretion between approximately 01.30 and 03.30. 

**Figure 3:**
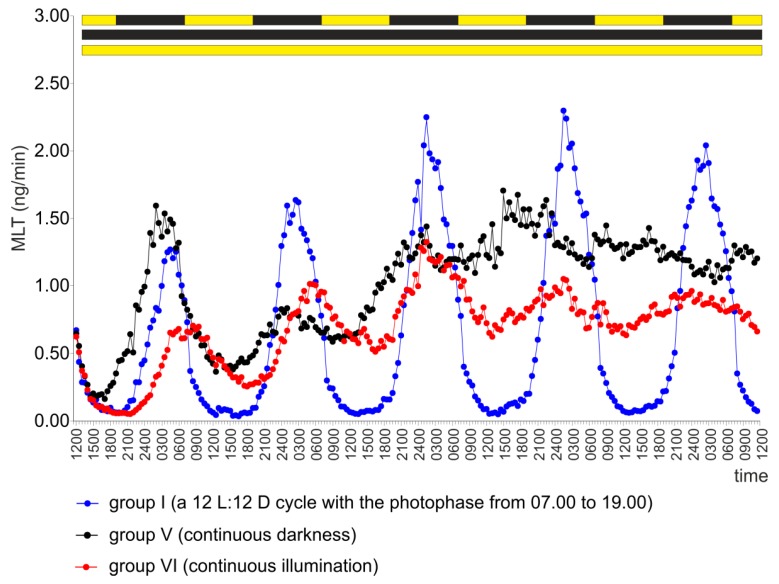
Experiment I. Secretion of MLT (mean) from turkey pineal organs incubated under a 12 L:12 D cycle with the photophase from 07.00 to 19.00 (group I), under continuous darkness (group V) or under continuous illumination (group VI).

The pineal organs incubated in continuous darkness (group V) showed circadian changes in MLT secretion throughout the experiment (Figure 3 and Figure S2). The amplitude of these changes was approximately 10-fold on the first day of culture but less than 2.5-fold on the following days. The minimal levels of MLT secretion were significantly higher, and the maximal levels of MLT secretion were significantly lower in the pineal organs incubated in continuous darkness than in the explants incubated under a 12 L:12 D cycle (Figure 3, Table 1). The circadian rhythm that occurred in continuous darkness showed a consecutive phase advance. To illustrate this result, the mid-points of the MLT peaks were located at 03.30 on day 1, at 01.05 on day 2, at 22.30 on day 3, at 20.30 on day 4 and at 13.10 on day 5 (Table 1).

The pineal organs incubated under continuous illumination (group VI) also secreted MLT with a circadian rhythm (Figure 3 and Figure S3). The amplitude of the MLT rhythm decreased gradually as the experiment progressed, mainly due to the increasing levels of the hormone on consecutive subjective day, with the amplitude varying from 13.6-fold on the first day to 1.5-fold on the last day. The occurrence of peaks was significantly delayed compared with group I during the first two days of the experiment (Table 1).

### 2.2. Experiment II

**Figure 4:**
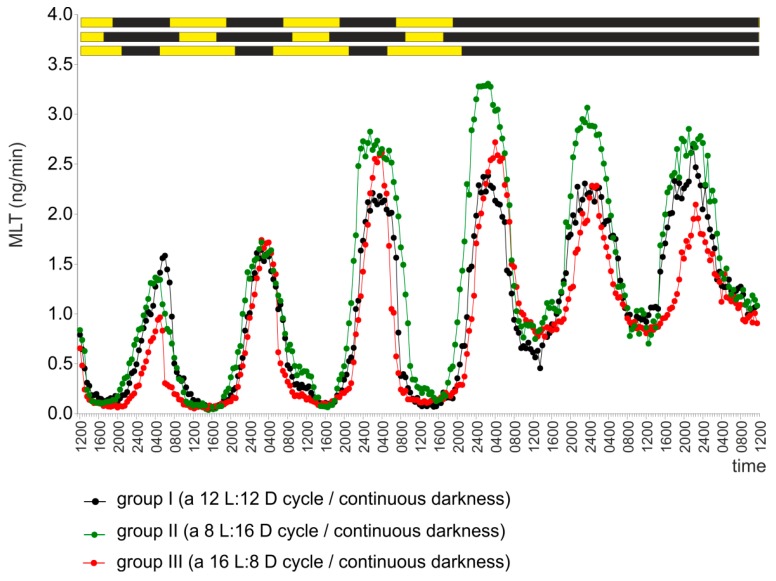
Experiment II. Secretion of MLT (mean) from turkey pineal organs incubated under a 12 L:12 D cycle (group I), a 8 L:16 D cycle (group II) and a 16 L:8 D cycle (group III) during four consecutive days and then in continuous darkness.

The pineal organs of group I secreted MLT in a regular rhythm during four days of incubation under a 12 L:12 D cycle (Figure 4). The duration of the nocturnal MLT peak, determined as described in the Materials and Methods section, varied between 13.1 and 13.9 hours (Table 2). The rhythmic patterns of MLT secretion persisted during the following two days of incubation in continuous darkness (Figure 4). The patterns of the MLT peaks generated during the subjective nights were similar to those occurring under a light-dark cycle, except for the lower amplitude. The duration of the MLT peak on the day 5 was 13.5 hours (Table 2). 

In the pineal organs (group II) incubated under a light-dark cycle with a 16-hour-long dark phase (8 L:16 D), the duration of elevated MLT secretion was fully entrained to the length of the scotophase starting from the second day of the experiment (Figure 4, Table 2). For example, the duration of the MLT peak on the first day of incubation was 13.6 hours, and that on the second day was 15.1 hours (Table 2). A circadian rhythm with prolonged periods of increased MLT secretion was observed during incubation in continuous darkness on day 5 of the experiment (peak duration 15.3 hours, Table 2). 

The secretion of MLT from the pineal organs (group III) incubated under a cycle with an 8-hour-long dark phase (16 L:8 D) occurred in a diurnal rhythm with periods of elevated nocturnal secretion that were shorter than those in groups I and II (Figure 4, Table 2). The duration of the MLT peak on the first day of culture was only 10.1 hours. On days 5 and 6, when the pineal organs were incubated in continuous darkness, the length of increased MLT secretion during the subjective nights was still shorter in this group than in the control group (Figure 4, Table 2). 

**Figure 5:**
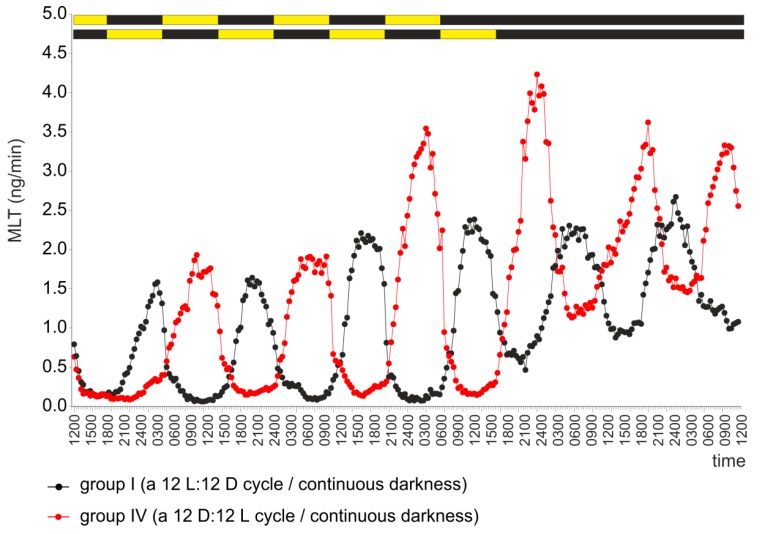
Experiment II. Secretion of MLT (mean) from turkey pineal organs incubated under a 12 L:12 D cycle (group I) and a reversed cycle 12 D:12 L (group III) during four consecutive days and then in continuous darkness.

The pineal organs (group IV) incubated during the first four days of the experiment under a 12 D:12 L cycle secreted MLT in a reversed rhythm starting from the second day of culture (Figure 5). The reversed MLT rhythm, whose amplitudes were reduced due to increased secretion during the subjective days, persisted during two days of incubation in continuous darkness (Figure 5).

### 2.3. Experiment III 

**Figure 6:**
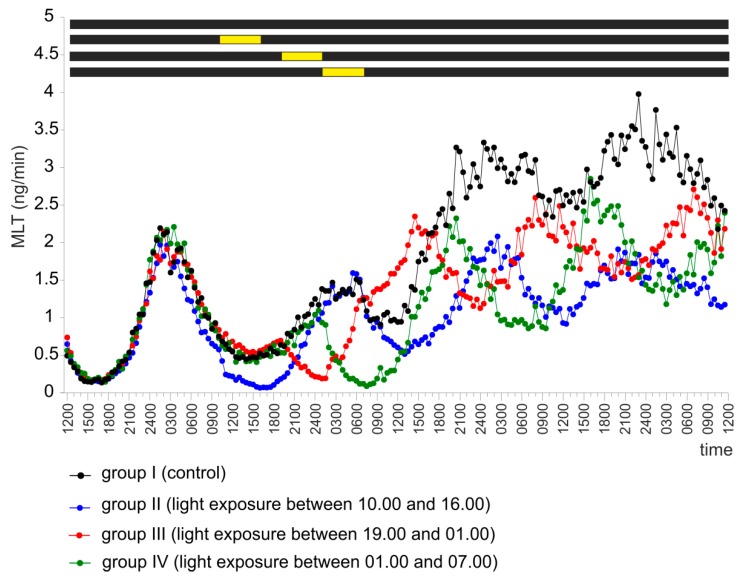
Experiment III. Secretion of MLT (mean) from turkey pineal organs incubated for four days in continuous darkness. During the second day of the experiment, the explants from groups II, III, and IV were exposed to fluorescent light with an intensity of 100 lx between 10.00 and 16.00 (group II), between 19.00 and 01.00 (group III), or between 01.00 and 07.00 (group IV).

The pineal organs incubated during four days in continuous darkness (group I) secreted MLT in the manner described for group V in experiment I (Figure 6). 

Exposure to light between 10.00 and 16.00 (group II), between 19.00 and 01.00 (group III) or between 01.00 and 07.00 (group IV) during the second day of the experiment resulted in a statistically significant decrease in MLT secretion (Figure 6 and Figure S4). The course of MLT secretion during the third and fourth days of the experiment was similar in unexposed explants and in the group of explants exposed to light between 10.00 and 16.00 (group II) (Figure 6 and Figure S5). Light exposure between 19.00 and 01.00 (group III) significantly delayed the occurrence of MLT peaks during days 3 and 4 of the experiment (Figure 6 and Figure S6). In contrast, light exposure between 01.00 and 07.00 (group IV) significantly advanced these endogenously generated peaks (Figure 6 and Figure S6).

### 2.4. Experiment IV 

**Figure 7:**
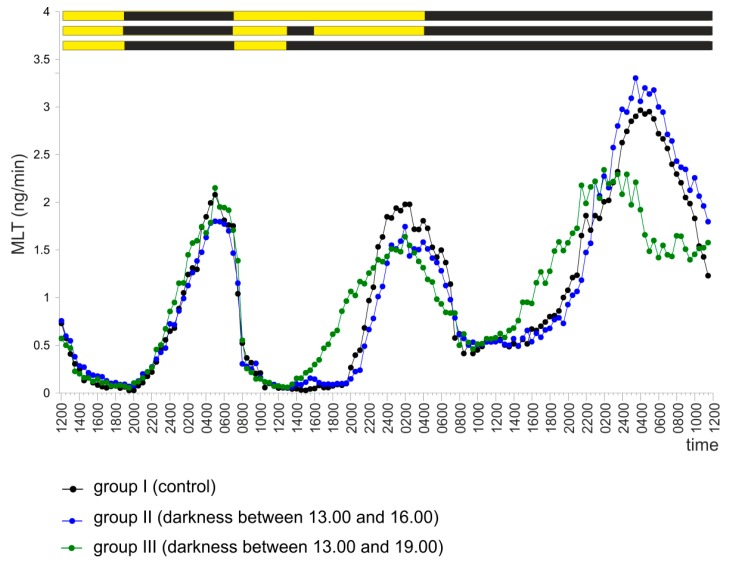
Experiment IV. Secretion of MLT (mean) from turkey pineal organs incubated under a 12 L:12 D cycle during the first two days of the experiment and then in continuous darkness for one day. The pineal organs from groups II and III were incubated in darkness between 13.00 and 16.00 (group II) or between 13.00 and 19.00 (group III) during the second day of the experiment. Group I (control) was not subjected to any additional treatment.

The pineal organs (group I) incubated under a 12 L:12 D cycle during the first two days of the experiment and then in continuous darkness for one day released MLT in a well-expressed rhythm (Figure 7). Incubation of the pineal organs in darkness between 13.00 and 16.00 during the second day (group II) resulted only in a small increase in the secretion of MLT. In contrast, incubation in darkness between 13.00 and 19.00 (group III) not only caused a larger increase in MLT secretion but also influenced the course of the MLT rhythm on the last day of the experiment (Figure 7 and Figure S7, Table S1).

### 2.5. Experiment V

**Figure 8:**
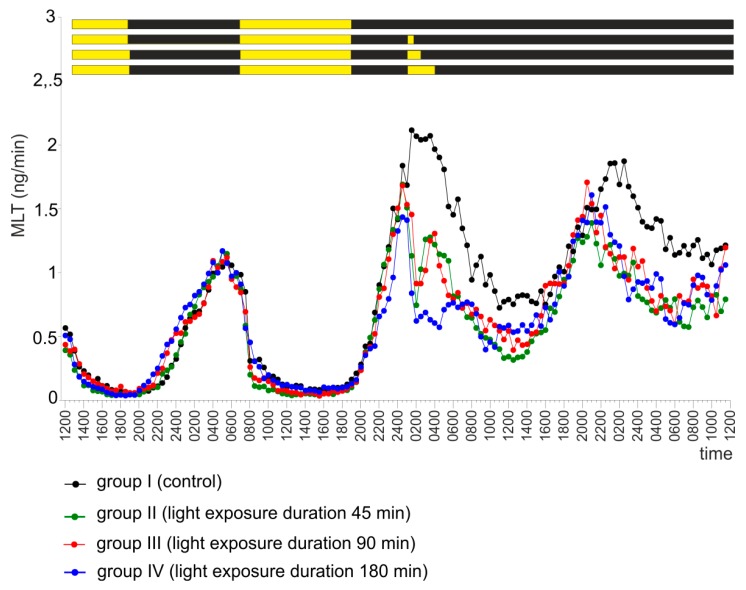
Experiment V. Secretion of MLT (mean) from turkey pineal organs incubated under a 12 L:12 D cycle during the first two days of the experiment and then in continuous darkness for one day. The pineal organs from groups II, III, and IV were exposed to light (fluorescent lamp, 100 lx) starting at 01.00 for 45 (group II), 90 (group III) or 180 (group IV) minutes during the second day of the experiment. Group I (control) was not subjected to any additional treatment.

The exposure of explants to light staring at 01.00 and lasting for 45, 90, or 180 minutes (groups II, III, and IV, respectively) during the second day of the experiment caused a significant decrease in MLT secretion (Figure 8 and Figure S8). In groups II and III, the secretion of MLT increased immediately after the end of light exposure. The largest decrease was observed after the 180-minute exposure (group IV). In all groups exposed to light, the peaks of MLT secretion on the last day of the culture were significantly advanced compared with group I (Table S2).

## 3. Discussion

Our results showed that turkey pineal organs incubated in superfusion culture under a 12 L:12 D cycle secrete MLT in a well-entrained, regular diurnal rhythm with low levels during the day and approximately 40-fold higher levels at night. The amplitude of this rhythm is much greater than the amplitude of MLT secretion rhythms in the pineal organs of other poultry species incubated in the same culture system. The difference between the highest and lowest levels of MLT secretion in vitro does not extend 10-fold in experiments involving the pineal organs of chickens [31,32]. The amplitudes of the diurnal rhythms of MLT secretion from the pineal organs of ducks (9 months old) and geese (12 weeks old) in superfusion culture were only 2-fold and 3-fold, respectively [7,25]. 

The turkey pineal gland is able to adjust its secretory activity extremely quickly and precisely to phase-shifts of light-dark cycles. The phases of MLT secretion rhythms were advanced or delayed by 6 hours or even reversed compared with the control group beginning on the second day of culture under the modified light-dark cycles. The adaptation of the pinealocytes of 15-week-old chickens, 12-week-old geese, and 9-month-old ducks to a reversed dark-light cycle takes more than two days [7,25,32]. Only the pineal organs of young chickens (3 weeks old) show a similar pattern to that observed in 12–14-week-old turkeys adapting to a new light schedule [31]. Additionally, the amplitudes of the MLT rhythms observed in turkey pineal organs cultured under advanced, delayed, or reversed cycles were close to those found under a natural light-dark cycle. Similar results have been reported only in experiments with the pineal organs of duck and goose nestlings [7,25]. Studies involving the pineal organs of 5- and 15-week-old chickens, 12-week-old geese, and adult ducks revealed a lower amplitude of MLT rhythms under the reversed cycle compared with the natural one [7,25,32]. 

Turkey pinealocytes precisely adapted their secretory activity to light-dark cycles reflecting natural cycles occurring in summer and winter. The period of elevated nocturnal MLT secretion was longer (in 8 L:16 D cycle) or shorter (16 L:8 D cycle) than those observed under a 12 L:12 D cycle beginning on the second day of incubation under a new light-dark cycle. There are no available data from similar in vitro studies in other avian species. Our results indicate the turkey pineal gland can provide precise information about the duration of the scotophase to other cells of the body via the secretion of MLT, thus acting as a biological calendar. 

In summary, the above experiments show that light acting directly on pinealocytes plays a crucial role in the regulation of MLT output in the turkey pineal organ. Comparison of the results obtained in our study with those from in vitro studies performed in other species strongly suggests that the turkey pineal organ shows the highest impact of direct photoreception on MLT secretion among the pineal organs of all examined domesticated birds. This observation agrees with the results of ultrastructural studies showing that pinealocytes of the rudimentary receptor type are the predominant type of pinealocytes in the pineal organ of 12–14-week-old turkeys and that they share many similarities in organization with the photoreceptor cells of fish pineal organs, except for the presence of an outer segment [45]. The rudimentary receptor pinealocytes of turkeys are much better developed than those of chickens, geese, and Muscovy ducks [37,38,42,48]. 

Relatively little is known about photopigments and signal transduction cascades mediating the effects of light in turkey pinealocytes. Several photopigments have been detected in the non-mammalian pineal organs including rhodopsin and iodopsin [52], parapinopisin [53], pinopsin [12,13], and melanopsin [14,54,55]. Our unpublished immunohistochemical study showed the presence of pinopsin in the pineal organs of 10-week-old turkeys. The pigment was localized in the apical extensions of rudimentary-receptor pinealocytes and short processes of secretory pinealocytes. Kang and co-workers [56] described the expression of melanopsin in the turkey pineal organ; however, the cellular distribution of this photopigment remains unknown.

The endogenous oscillatory property of the avian pineal gland was first proved by in vitro studies in dispersed chick pineal cell cultures, which able to maintain circadian oscillations of MLT secretion for 2 cycles in constant darkness [57]. Subsequently, endogenously generated circadian rhythms of pineal MLT release in birds were demonstrated in isolated single cells [30] and cultured organs [7,23,25,31,32]. To check whether MLT secretion rhythm in the turkey is generated endogenously, the pineal glands were incubated in constant darkness and constant light. It is known that light inhibits MLT production from the pineal organ, so it can be expected that the MLT secretion under constant light will remain at a constant very low level. Contrary to our expectations, the turkey pineal organs secreted MLT in circadian rhythms under both 0 L:24 D and 24 L:0 D conditions for 4–5 days. The phases of the rhythm were shifted forward in continuous darkness and backward in continuous illumination compared with those under the 12 L:12 D cycle. The amplitude of the rhythm decreased in consecutive cycles. Studies on other avian pineal glands incubated in constant darkness revealed the presence of species variability in the activity of pineal oscillators. In pigeon and sparrow pineal glands, the rhythm of MLT release persists for 3–4 days [58]. In contrast, the circadian rhythm of MLT secretion from the pineal organs of Japanese quails in vitro was found to be weak or completely abolished, while it was maintained in vivo in continuous darkness [58,59]. These data suggest that extrapineal oscillators play a crucial role in the generation of the MLT synthesis rhythm in this species. The circadian rhythm of plasma MLT in young turkeys maintained in continuous darkness persisted for seven days [4,60]. A longer-lasting in vivo MLT rhythm may result from the influence of oscillators located outside the pineal gland. However, the effect of the age of birds should also considered because age-dependent differences in the activity of the pineal oscillator have been described in *Anseriformes* [7,25,61]. The circadian rhythm of MLT secretion in vitro in constant darkness was well expressed in 1-day-old ducks while in 9-month-old ducks, it was characterized by a very small amplitude [25]. 

The most important and promising results were obtained in the experiments in which the incubation of the pineal organs under various light-dark cycles was followed by incubation in continuous darkness. The pineal organs that were incubated under 12 L:12 D, 16 L:8 D, 8 L:16 D, and 12 D:12 L cycles and then placed in continuous darkness secreted MLT in a circadian rhythm in which the period of increased secretion corresponded to the scotophase of previous light-dark cycles. This result shows that the endogenous oscillator of turkey pinealocytes adapts to changing light conditions very easily in vitro, stores information about the last cycle, and generates a cycle according the acquired data under continuous culture conditions. The phenomenon of the pineal gland learning a new light schedule has been previously reported in the house sparrow [23,58]. 

To analyze the response of the turkey pineal organ to light, we exposed explants to unexpected light during various phases of night. All light pulses caused a significant decrease in MLT secretion, but it should be emphasized that it was only the 6-hour exposure decreased the MLT level to the daytime level. The in vivo inhibitory effect of light in the turkey was stronger than that in vitro, which suggests the involvement of retina photoreception in the regulation of the secretory activity of the turkey pineal gland; however, the possibility of age-dependent differences cannot be excluded [4,5,60]. Light exposure influenced the activity of the turkey pineal oscillator and induced a phase-shift in the circadian MLT rhythm. This effect was strictly dependent on the time of exposure. All light pulses applied in the first half of the night delayed the occurrence of successive peaks of MLT secretion, while those in the second half of the night advanced the occurrence of successive peaks of MLT secretion in continuous darkness. The phase-shifting effect of light was well characterized in the chicken pineal organ [62,63,64,65,66]

Based on studies conducted in chicken, it has been suggested that two distinct effects of light on the pineal organ—the acute suppression of MLT synthesis and the phase shift in endogenous oscillator activity—are mediated by two different photopigments: Pinopsin and melanopsin [12,14]. Pinopsin is probably responsible for the inhibitory action of light on MLT secretion, while melanopsin is considered to be involved in the functionality of the intrapineal oscillator. The mechanisms responsible for the variable light effects on endogenous oscillator activity in the first and the second half of the night are unknown. One of many hypotheses assumes the presence of “morning” and “evening” clock components in the endogenous oscillator [67]. 

Interestingly, the turkey pineal organs exposed to light in the middle of the subjective day while being cultured in continuous darkness (between 10.00 and 16.00) showed the same later course of the MLT rhythm observed in the control group. This result shows that the sensitivity of the adaptation mechanisms of the endogenous oscillator is restricted to the night. Light stimuli during the subjective night interfere with the “stored” rhythm, thus providing information about changes in night length and leading to modification of oscillator activity. In turn, light exposure during the subjective day, corresponding to the memorized photophase of the previous cycle, did not cause significant changes in the functionality of the endogenous generator. 

To complete our research, in addition to the light pulses at scotophase, we also examined the effect of periods of darkness on the activity of the pineal oscillator in the turkey. Darkness lasting 3 or 6 hours applied during photophase caused a gradual increase in MLT secretion. However, only a 6-hour-long period of darkness used in the second half of the day resulted in the occurrence of the advanced MLT peak. The experiment involving exposure to darkness during the day is the first to be performed in vitro on the avian pineal gland. 

## 4. Materials and Methods 

### 4.1. Chemicals

Medium 199 containing Earle’s salt and HEPES (Sigma-Aldrich, St. Louis, MO, USA) was prepared from a powder according the manufacturer’s instructions (pH adjusted to 7.2 with NaOH) and sterilized by filtration. An antibiotic-antimycotic solution (Sigma-Aldrich, St. Louis, MO, USA) containing penicillin (100 IU/ml), streptomycin (100 μg/ml), and amphotericin B (0.25 μg/ml) was added to the medium immediately before use. 

The anti-melatonin antibody Prospect 6C was kindly provided by Dr Andrew Foldes (Division of Animal Production, CSIRO, Blacktown, Australia); ^3^H-melatonin was purchased from PerkinElmer (Waltham, MA, USA), gelatin from Merck (Billerica, MA, USA), toluene from POCH, Gliwice Poland. All other reagents used in melatonin RIAs were from Sigma (St. Louis, MO, USA). 

### 4.2. Animals and Pineal Organs

The pineal organs were obtained from female turkeys aged 12–14 weeks (body weight 7.5 ± 0.8 kg). The birds were kept under a cycle with 12-hour-long photophase (from 07.00 to 19.00) and 12-hour-long scotophase, starting from the 6th week of postembryonic life. During the photophase, fluorescent lamps provided light with an intensity of 100 lx at the level of the bird’s heads. Red light with an intensity of less than 10 lx was used during the scotophase. The animals had free access to standard food and water. The turkeys were sacrificed at 8.00, their pineal glands were immediately removed, placed into the culture medium and transported to the cell culture lab within less than 5 minutes. All experimental procedures on animals were performed in accordance with Polish and EU law.

### 4.3. Superfusion Culture

The pineal organs were covered with nylon mesh and placed in separate culture chambers (volume 0.5 ml). The lower pool of each chamber was connected to the container with culture medium. The upper pool of the culture chambers was attached to a multichannel peristaltic pump (Cole Parmer, Vernon Hills, IL, USA) and a manual fraction collector. The total volume of the superfusion set consisting of the culture chamber and tubes was 1.3–1.4 ml. Superfusion was performed at a flow rate of 0.1 ml/min. The medium was continuously gassed with a mixture of 95% O_2_ and 5% CO_2_. Incubation was performed at 38.5 °C in a water bath (Julabo, Seelbach, Germany). The culture chambers were covered with translucent plastic sheets during incubation in light and with similar light-proof sheets during incubation in darkness. The chambers were illuminated with a full-spectrum fluorescent lamp providing light with an intensity of 100 lx at the surface of the sheets covering the perfusion chambers. Medium fractions were collected every 30 minutes on consecutive days and frozen at −20 °C until the melatonin assay was performed. The time-point of 07.00 was taken as the beginning of day 2 and the following days.

### 4.4. Experimental Procedures

#### 4.4.1. Experiment I 

The experiment was performed on six groups of pineal organs (4 glands per group) cultured under different light conditions for a period of five consecutive days. The pineal organs of group I were incubated under a light-dark cycle with the photophase from 07.00 to 19.00 (12 L:12 D). The pineal organs of group II were exposed to a cycle with an advanced photophase from 01.00 to 13.00 and those of group III to a cycle with a delayed photophase from 13.00 to 01.00. Group IV was incubated under a reversed dark-light cycle with the photophase from 19.00 to 07.00 (12 D:12 L). The pineal organs of group V were kept in continuous darkness, and those of group VI under continuous illumination.

#### 4.4.2. Experiment II 

The experiment was conducted during six consecutive days. The chambers holding the pineal organs of the turkeys were randomly assigned to one of four groups (4 glands per group). Group I of the explants (control) was incubated under a light-dark cycle with the photophase from 07.00 to 19.00 during the first four days (12 L:12 D). At the same time, group II was cultured under a cycle with an 8-hour photophase from 09.00 to 17.00 (8 L:16 D); group III, under a cycle with 16-hour photophase from 05.00 to 21.00 (16 L:8 D); and group IV, under a reversed dark-light cycle with the photophase from 19.00 to 07.00 (12 D:12 L). During the last two days of the experiment, the pineal organs of all four groups were incubated under continuous darkness.

#### 4.4.3. Experiment III 

The experiment was performed on four groups of pineal organs (4 glands per group) that were incubated for four days in continuous darkness. On the second day of the experiment, the pineal organs were exposed to fluorescent light with an intensity of 100 lx between 10.00 and 16.00 (group II), between 19.00 and 01.00 (group III) or between 01.00 and 07.00 (group IV). The explants of group I served as a control and remained unexposed.

#### 4.4.4. Experiment IV 

The experiment was performed in three groups of pineal organs (4 glands per group) that were incubated under a 12 L:12 D cycle during the first two days of the experiment and then in continuous darkness for one day. Group I (control) was not subjected to any additional treatment. The pineal organs of groups II and III were incubated in darkness between 13.00 and 16.00 (group II) or between 13.00 and 19.00 (group III) during the second day of the experiment. 

#### 4.4.5. Experiment V 

The experiment was performed in four groups of pineal organs (4 glands per group) that were incubated under a 12 L:12 D cycle during the first two days of the experiment and then in continuous darkness for one day. Group I (control) was not subjected to any additional treatment. The pineal organs from groups II, III and IV were exposed to light (fluorescent lamp, 100 lx) beginning at 01.00 for 45 (group II), 90 (group III), or 180 (group IV) minutes during the second day of the experiment.

### 4.5. Melatonin Radioimmunoassay 

The MLT concentration in medium samples was measured using direct RIA [68] with Prospect 6 C antiserum [69] and ^3^H-melatonin (3.2 TBq/mmol). Briefly, 200 μl of antiserum diluted 1:100000 in assay buffer (tricine 0.1 mmol/L, sodium chloride 9 g/L, and gelatin 1g/L) was added to test tubes containing 100 μl of assay buffer (or standards in tubes for calibration curve) and 20 μl of sample (or pure medium in tubes for calibration curve). The mixture was incubated at room temperature for 30 min., and then 100 μl of ^3^H-melatonin solution in assay buffer (approximately 15000 dpm) was added. After overnight incubation at 4° C, bound melatonin was separated from the free one by incubation with 250 μl of dextran coated charcoal (0.6 g of Norit A and 60 mg of dextran in 100 ml assay buffer) at 4 °C for 20 min. and centrifugation (3000 G, 20 min., 4 °C, Beckman J-6 centrifuge, Beckman, Pasadena, CA, USA). Next, 350 μL of supernatant was transferred to scintillation vial, 4 ml of scintillator (5 g PPO and 0.3 g dimethyl-POPOP in 1 L toluene) was added and the radioactivity was measured in LS 6500 Scintillator Counter (Beckman, Pasadena, CA, USA). The concentration of MLT in samples was determined using ImmunoFit EIA/RIA ver. 3.0A software (Beckman, Pasadena, CA, USA). The calibration curve was characterized by an equation y = (A−D)/1+(x/C)^B)+D, in which A = 1380 ± 180, B = 0.95 ± 0.07, C = 45.7 ± 0.04, and D = −1.2 ± 0.3, *r* = 0.999 (mean ± SEM, *n* = 15). Specific binding was 36.8 ± 5% and unspecific binding 0.9 ± 0.2 (mean ± SEM, *n* = 15). The intra- and inter-assay coefficients of variation were below 10%. The assay was validated by comparison of MLT concentrations measured in the same medium samples by RIA and by HPLC with fluorescence detection [70,71]. The correlation between these methods was very high (y = 1.03x+1.92; *R* = 0.999, *p* < 0.000001). 

### 4.6. Statistical Analysis

The data on the concentration of MLT in medium samples were analyzed using repeated measures analysis of variance (ANOVA) with the treatment as the main effect and the sampling time as a repeated measure. The least significant difference test (LSD) was applied as a post hoc procedure. 

To assess the changes in the course of the MLT secretion rhythm, individual profiles of MLT concentrations in medium samples were plotted, and the following points were determined in each cycle: (1) Minimum-parameters of the point (value and daily time) with the lowest concentration of MLT; (2) maximum-parameters of the point (value and daily time) at which secretion reached the maximum value; (3) I_50—_the point (daily time) at which secretion increased to 50% of the maximum value; (4) I_75—_the point (daily time) at which secretion increased to 75% of the maximum value; (5) D_50—_the point (daily time) at which secretion decreased to 50% of the maximum value; (6) D_75—_the point (daily time) at which secretion decreased to 75% of the maximum value; (7) M (the middle of the peak)—the point (daily time) halfway between the I_75_ and D_75_ points. When MLT secretion was investigated under continuous darkness and continuous light, it was not possible to determine the D_50_ point because the decrease in MLT secretion was mostly lower than 50%. Thus, for determination of the M point values, the I_75_ and D_75_ points were used. These data were analyzed using a one-way analysis of variance followed by Duncan’s test. To perform these calculations, minutes were expressed as tenths of hours (e.g., 15:15 = 15.25). 

Additionally, in experiment II, the duration of increased MLT secretion during the scotophase or the subjective night was calculated as the period between the first point, when the concentration of MLT differed significantly from the lowest value in the previous photophase or on the previous subjective day, and the last point, when the concentration of MLT differed significantly from the lowest value in the following photophase or on the following subjective day. Both time-points were determined based on the results of the LSD test performed as a post hoc procedure of repeated measures ANOVA. The obtained values for the duration of increased MLT secretion during the scotophase or the subjective night were analyzed using one-way analysis of variance followed by Duncan’s test. 

Statistical analyses were performed using Statistica 6.0 PL (StatSoft Polska, Cracow, Poland) and SPSS 11.0 (SPSS Inc., Chicago, Il, USA) working in Windows XP (Microsoft, Redmond, WA, USA).

## 5. Conclusions

The turkey pineal organ in superfusion culture is characterized by an extremely well-entrained diurnal rhythm of MLT secretion with a very high amplitude compared to the pineal organs of other examined domesticated birds. Direct photoreception as an independently acting mechanism ensures quick and precise adaptation of the MLT secretion rhythm in the turkey pineal organ to changes in light-dark conditions. This situation is different than those in ducks, geese, and chickens of the same age. In superfusion culture, the endogenous oscillator of turkey pinealocytes acquires and stores information about the light-dark cycle and generates the circadian rhythm of MLT secretion in continuous darkness according to the stored data. The turkey pineal organ secretes MLT in a circadian rhythm even when culture is performed under continuous light. Our data show that the turkey pineal gland can be highly autonomous in the generation and regulation of the rhythm of MLT secretion. The obtained results are important in terms of comparative endocrinology, as they reveal prominent interspecies differences. However, they also demonstrate that the turkey pineal organ in superfusion culture is a valuable model for chronobiological studies, providing a highly precise clock and calendar. This system has several features which make it an attractive alternative to other avian pineal glands for circadian studies.

## Figures and Tables

**Table 1:** Parameters (mean ± SEM) characterizing the rhythm of MLT secretion from the pineal organs in experiment I. The minimum and maximum points are characterized by the time clock presented in the format “hh.mm” (the standard error in minutes) and the level of secretion in ng/min. Points I_50_ and I_75_ and M are expressed in units of the time clock in the format “hh.mm” (the standard error in minutes). The values labeled with different letters differ significantly (between groups) by the Duncan test (*p* ≤ 0.05). n.d.—not determined.

Groups	Minimum		Maximum		I_50_	I_75_	M
	Clock Time	Secretion Level	Clock Time	Secretion Level			
**Day I**							
Group I 12 L:12 D	18.15 ^a^ ( ± 25.1)	0.06 ^a^ ( ± 0.01)	04.32 ^a^ ( ± 20.2)	1.27 ^a^ ( ± 0.25)	00.37 ^a^ ( ± 10.7)	03.08 ^a^ ( ± 21.7)	04.32 ^a^ ( ± 11.4)
Group II 12 L:12 D ^1^	18.07 ^a^ ( ± 37.5)	0.13 ^a b^ ( ± 0.05)	01.04 ^d^ ( ± 5.7)	1.31 ^a^ ( ± 0.12)	23.30 ^d^ ( ± 25.63)	00.08 ^d^ ( ± 15.7)	n.d.
Group III 12 L:12 D ^2^	22.17 ^b^ ( ± 32.7)	0.04 ^a^ ( ± 0.01)	07.00 ^c^ ( ± 15.2)	1.37 ^a^ ( ± 0.07)	03.37 ^e^ ( ± 32.5)	05.09 ^c^ ( ± 42.5)	07.10 ^c^ ( ± 15.5)
Group IV 12 D:12 L	n.d.	n.d.	n.d.	n.d.	n.d.	n.d.	n.d.
Group V 0 L:24 D	17.07 ^a^ ( ± 34.5)	0.16 ^b^ ( ± 0.02)	02.08 ^b^ ( ± 32.7)	1.58 ^a^ ( ± 0.23)	21.32 ^b^ ( ± 21.4)	01.14 ^b^ ( ± 17.3)	03.30 ^b^ ( ± 12.4)
Group VI 24 L:0 D	21.28 ^b^ ( ± 21.5)	0.05 ^a^ ( ± 0.01)	06.17 ^c^ ( ± 41.2)	0.68 ^b^ ( ± 0.09)	02.07 ^c^ ( ± 32.1)	04.10 ^c^ ( ± 21.4)	07.00 ^c^ ( ± 10.2)
**Day II**							
Group I 12 L:12 D	12.08 ^a^ ( ± 40.8)	0.04 ^a^ ( ± 0.01)	02.37 ^a^ ( ± 19.0)	1.63 ^a^ ( ± 0.24)	23.08 ^a^ ( ± 19.7)	00.17 ^a^ ( ± 22.7)	02.38 ^a^ ( ± 23.4)
Group II 12 L:12 D ^1^	10.32 ^c^ ( ± 55.8)	0.09 ^a^ ( ± 0.01)	21.10 ^c^ ( ± 35.5)	1.98 ^a^ ( ± 0.21)	16.40 ^c^ ( ± 42.5)	18.15 ^c^ ( ± 33.7)	21.12 ^d^ ( ± 26.7)
Group III 12 L:12 D ^2^	20.07 ^d^ ( ± 25.7)	0.05 ^a^ ( ± 0.01)	07.28 ^d^ ( ± 32.5)	1.49 ^a^ ( ± 0.04)	04.09 ^d^ ( ± 25.4)	06.10 ^d^ ( ± 35.7)	08.00 ^e^ ( ± 14.7)
Group IV 12 D:12 L	01.32 ^e^ ( ± 41.2)	0.14 ^a^ ( ± 0.01)	14.02 ^e^ ( ± 17.8)	1.70 ^a^ ( ± 0.23)	08.31 ^e^ ( ± 23.5)	10.14 ^e^ ( ± 44.2)	13.12 ^f^ ( ± 55.4)
Group V 0 L:24 D	12.29 ^a^ ( ± 21.4)	0.36 ^b^ ( ± 12.7)	01.02 ^a^ ( ± 24.9)	0.83 ^b^ ( ± 11.2)	18.07 ^b^ ( ± 11.4)	20.08 ^b^ ( ± 24.3)	01.05 ^b^ ( ± 17.8)
Group VI 24 L:0 D	18.19 ^b^ ( ± 25.8)	0.27 ^b^ ( ± 0.03)	05.12 ^b^ ( ± 31.2)	1.01 ^b^ ( ± 0.02)	23.39 ^a^ ( ± 19.8)	01.29 ^a^ ( ± 37.8)	05.10 ^c^ ( ± 24.9)
**Day III**							
Group I 12 L:12 D	13.11 ^a^ ( ± 19.7)	0.05 ^a^ ( ± 0.01)	01.37 ^a^ ( ± 28.4)	2.23 ^a^ ( ± 0.42)	22.37 ^a^ ( ± 39.3)	23.34 ^a^ ( ± 29.5)	02.28 ^a^ ( ± 38.4)
Group II 12 L:12 D ^1^	09.07 ^b^ ( ± 27.1)	0.08 ^a^ ( ± 0.01)	20.27 ^b^ ( ± 41.2)	2.19 ^a^ ( ± 0.21)	16.10 ^c^ ( ± 21.4)	17.35 ^b^ ( ± 25.7)	21.00 ^b^ ( ± 32.78)
Group III 12 L:12 D ^2^	20.32 ^d^ ( ± 24.5)	0.06 ^a^ ( ± 0.01)	08.07 ^c^ ( ± 48.7)	1.49 ^a b^ ( ± 0.41)	04.10 ^d^ ( ± 15.2)	05.40 ^c^ ( ± 22.3)	07.45 ^c^ ( ± 18.7)
Group IV 12 D:12 L	01.21 ^e^ ( ± 25.7)	0.12 ^a^ ( ± 0.02)	14.48 ^d^ (±24.9)	2.87 ^a^ ( ± 0.12)	09.27 ^e^ ( ± 14.7)	11.07 ^d^ ( ± 24.7)	14.05 ^d^ ( ± 19.8)
Group V 0 L:24 D	09.05 ^b^ ( ± 22.5)	0.59 ^b^ ( ± 0.09)	01.30 ^a^ ( ± 19.8)	1.44 ^b^ ( ± 12.5)	13.55 ^b^ ( ± 22.3)	17.05 ^b^ ( ± 12.9)	22.30 ^b^ ( ± 42.5)
Group VI 24 L:0 D	16.29 ^c^ ( ± 25.6)	0.52 ^b^ ( ± 0.04)	01.35 ^a^ ( ± 25.5)	1.32 ^b^ ( ± 0.13)	19.37 ^a^ ( ± 42.3)	22.14 ^a^ ( ± 31.89)	03.08 ^a^ ( ± 28.1)
**Day IV**							
Group I 12 L:12 D	13.07 ^a^ ( ± 27.4)	0.05 ^a^ ( ± 0.02)	01.35 ^a^ ( ± 17.8)	2.29 ^a^ ( ± 0.22)	22.37 ^a^ ( ± 36.2)	00.07 ^a^ ( ± 25.6)	02.28 ^a^ ( ± 22.2)
Group II 12 L:12 D ^1^	09.08 ^b^ ( ± 21.4)	0.11 ^a^ ( ± 0.04)	19.02 ^c^ ( ± 25.7)	2.52 ^a^ ( ± 0.21)	15.07 ^c^ ( ± 31.2)	17.05 ^d^ ( ± 14.5)	20.01 ^b^ ( ± 25.7)
Group III 12 L:12 D ^2^	21.08 ^d^ ( ± 21.4)	0.05 ^a^ ( ± 0.01)	08.29 ^d^ ( ± 32.8)	1.38 ^a b^ ( ± 0.31)	04.32 ^d^ ( ± 23.5)	05.17 ^e^ ( ± 42.5)	08.32 ^d^ ( ± 32.5)
Group IV 12 D:12 L	03.28 ^e^ ( ± 27.8)	0.11 ^a^ ( ± 0.05)	15.05 ^e^ ( ± 42.5)	2.79 ^a^ ( ± 0.07)	10.07 ^e^ ( ± 23.5)	11.25 ^f^ ( ± 17.5)	14.17 ^e^ ( ± 25.7)
Group V 0 L:24 D	09.55 ^b^ ( ± 42.5)	1.09 ^b^ ( ± 0.23)	17.24 ^b^ ( ± 19.8)	1.65 ^b^ ( ± 0.17)	n.d.	15.04 ^b^ ( ± 27.8)	20.03 ^b^ ( ± 19.8)
Group VI 24 L:0 D	13.21 ^c^ ( ± 21.7)	0.65 ^b^ ( ± 0.19)	02.10 ^a^ ( ± 32.5)	1.04 ^b^ ( ± 0.2)	18.32 ^b^ ( ± 32.5)	21.14 ^c^ ( ± 22.7)	23.51 ^c^ ( ± 23.2)
**Day V**							
Group I 12 L:12 D	13.28 ^a^ ( ± 21.2)	0.06 ^a^ ( ± 0.01)	02.57 ^a^ ( ± 18.4)	2.03 ^a^ ( ± 0.17)	22.00 ^a^ ( ± 24.2)	23.32 ^a^ ( ± 14.7)	02.41 ^a^ ( ± 19.7)
Group II 12 L:12 D ^1^	06.07 ^c^ ( ± 25.8)	0.09 ^a^ ( ± 0.01)	20.04 ^d^ ( ± 34.7)	1.97 ^a^ ( ± 21.4)	16.10 ^b^ ( ± 29.8)	17.17 ^d^ ( ± 17.8)	20.01 ^c^ ( ± 20.7)
Group III 12 L:12 D ^2^	21.04 ^d^ ( ± 55.2)	0.05 ^a^ ( ± 0.01)	08.30 ^b^ ( ± 32.5)	1.25 ^a b^ ( ± 0.42)	04.10 ^c^ ( ± 24.8)	05.30 ^e^ ( ± 32.1)	07.38 ^d^ ( ± 35.5)
Group IV 12 D:12 L	02.30 ^e^ ( ± 24.9)	0.17 ^a^ ( ± 0.03)	15.01 ^e^ ( ± 12.5)	2.08 ^a^ ( ± 0.21)	10.05 ^d^ ( ± 32.5)	10.55 ^f^ ( ± 42.5)	14.02 ^b^ ( ± 32.5)
Group V 0 L:24 D	04.09 ^b^ ( ± 23.5)	1.02 ^b^ ( ± 012)	09.01 ^b^ ( ± 42.3)	1.44 a ^b^ ( ± 15.7)	n.d.	07.08 ^b^ ( ± 33.5)	13.10 ^b^ ( ± 25.8)
Group VI 24 L:0 D	12.35 ^a^ ( ± 32.5)	0.63 ^b^ ( ± 0.04)	00.14 ^c^ ( ± 23.5)	0.96 ^b^ ( ± 28.9)	n.d.	19.05 ^c^ ( ± 19.8)	01.07 ^a^ ( ± 26.7)

^1^ Photophase from 01.00 to 13.00, ^2^ Photophase from 13.00 to 01.00.

**Table 2:** Duration of increased MLT secretion during the scotophase or subjective night (mean, time clock presented in the format “hh.mm”; the standard error in minutes). The values labeled with different letters differ significantly (between groups) by the Duncan test (*p* ≤ 0.05). n.d.—not determined.

Groups	Day I	Day II	Day III	Day IV	Day V	Day VI
Group I 12 L:12 D	13.6 ^A^ ( ± 23.2)	13.5 ^A^ ( ± 18.6)	13.6 ^A^ ( ± 14.8)	13.9 ^A^ ( ± 16.1)	13.5 ^A^ ( ± 14.9)	13.1 ^A^ ( ± 19.2)
Group II 16 L:8 D	10.1 ^B^ ( ± 24.9)	11.2 ^B^ ( ± 14.9)	10.4 ^B^ ( ± 15.6)	11.7 ^B^ ( ± 14. 1)	11.1 ^B^ ( ± 15.7)	11.5 ^B^ ( ± 18.9)
Group III 8 L:16 D	13.6 ^A^ ( ± 20.4)	15.1 ^C^ ( ± 21.8)	15.5 ^C^ ( ± 21.7)	14.7 ^C^ ( ± 15.1)	15.2 ^C^ ( ± 19.2)	13.2 ^A^ ( ± 22.5)
Group IV 12 D:12 L	n.d.	13.5 ^A^ ( ± 19.5)	13.7 ^A^ ( ± 15.7)	14.1 ^A^ ( ± 14.8)	13.5 ^A^ ( ± 14.9)	14.2 ^A^ ( ± 20.4)

## Supplementary Materials

Supplementary materials can be found at https://www.mdpi.com/1422-0067/20/16/4022/s1.
