# Supplementary material for: Roles of Direct Photoreception and the Internal Circadian Oscillator in the Regulation of Melatonin Secretion in the Pineal Organ of the Domestic Turkey: A Novel In Vitro Clock and Calendar Model
Source: Int J Mol Sci. 2019 Aug 17;20(16):4022. doi: 10.3390/ijms20164022 (PMC6721154; doi:10.3390/ijms20164022)
Supplement: Supplementary file 1 [file ijms-20-04022-s001.pdf]

Figure S1 Statistical evaluation of time-dependent changes in MLT secretion from turkey pineal organs incubated under a 12 L:12 D cycle with the photophase from 07.00 to 19.00 (group I) in experiment I. The data shown are mean and SEM. Values labeled with different letters are significantly different (p≤0.05).

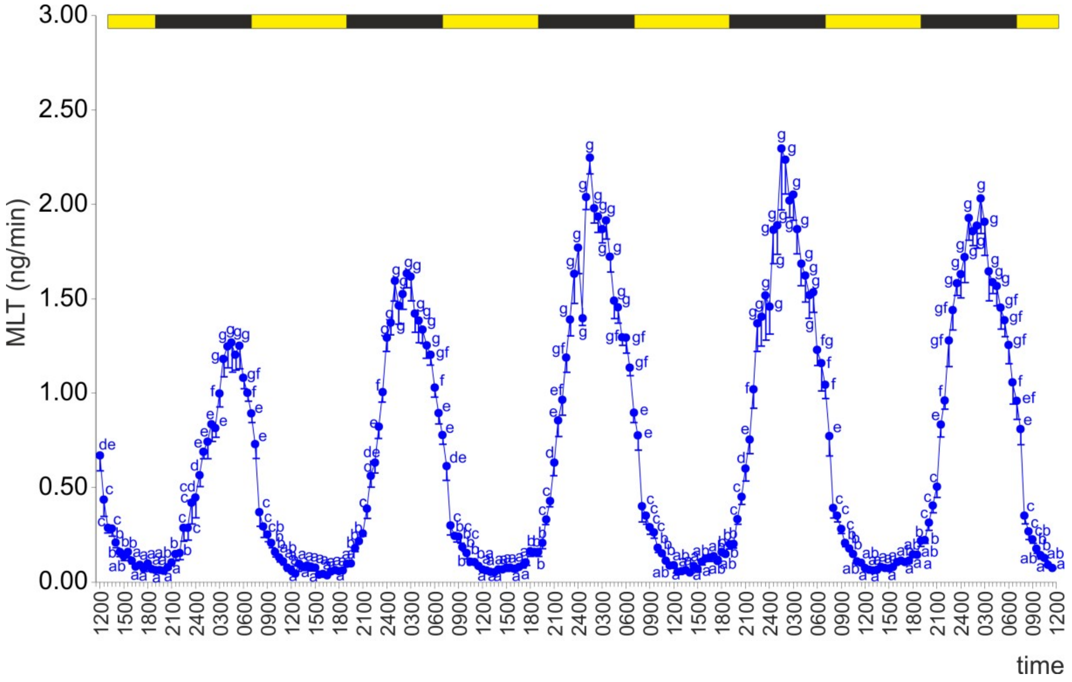

Figure S2. Individual profiles of MLT secretion from pineal organs incubated in continuous darkness.

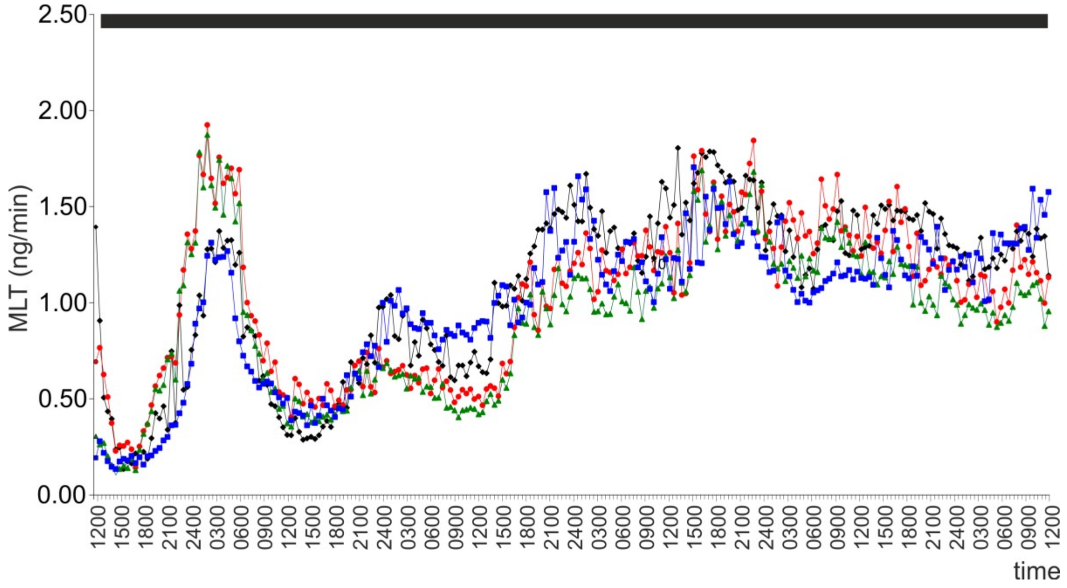

Figure S3. Individual profiles of MLT secretion from pineal organs incubated in continuous illumination.

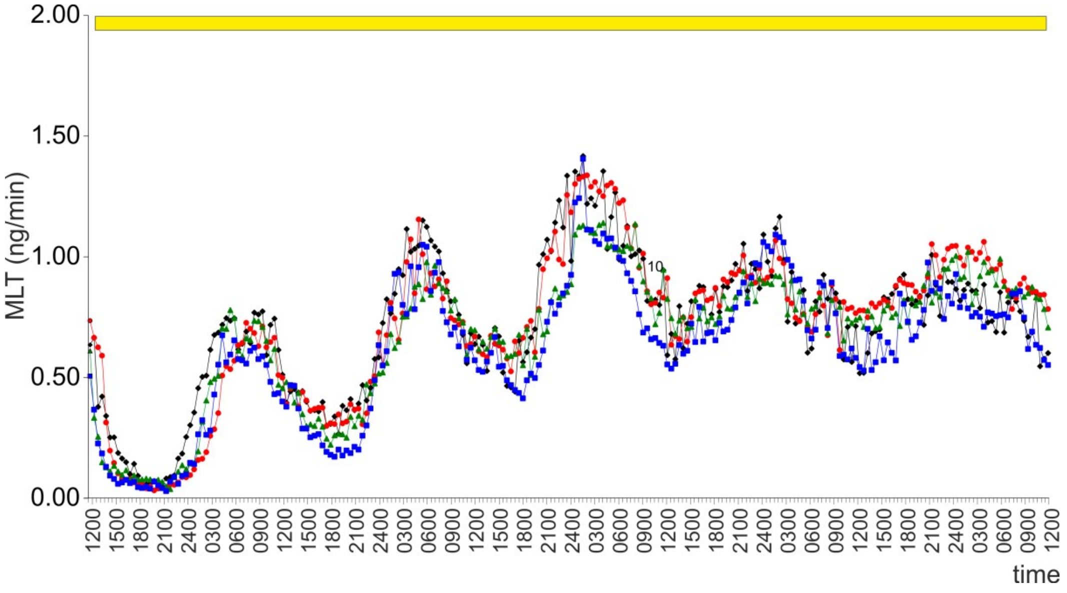

Figure S4. Inhibitory effect of light exposure on MLT secretion (mean and SEM) from the pineal organs of group II – IV in experiment III. Values labeled with different letters differ significantly (p≤0.05) between groups.

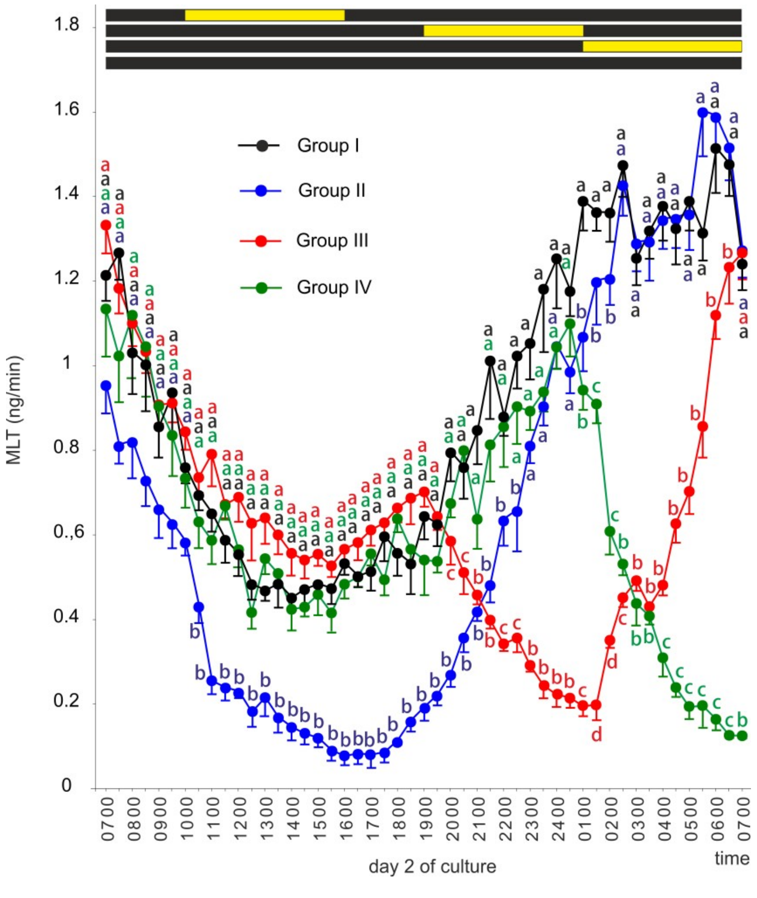

Figure S5. Lack of effect of light exposure during the subjective day on the phase of circadian rhythm of MLT secretion during days III and IV (group II, experiment III). The midway points of the peaks (clock time, mean and SEM) are marked with vertical lines.

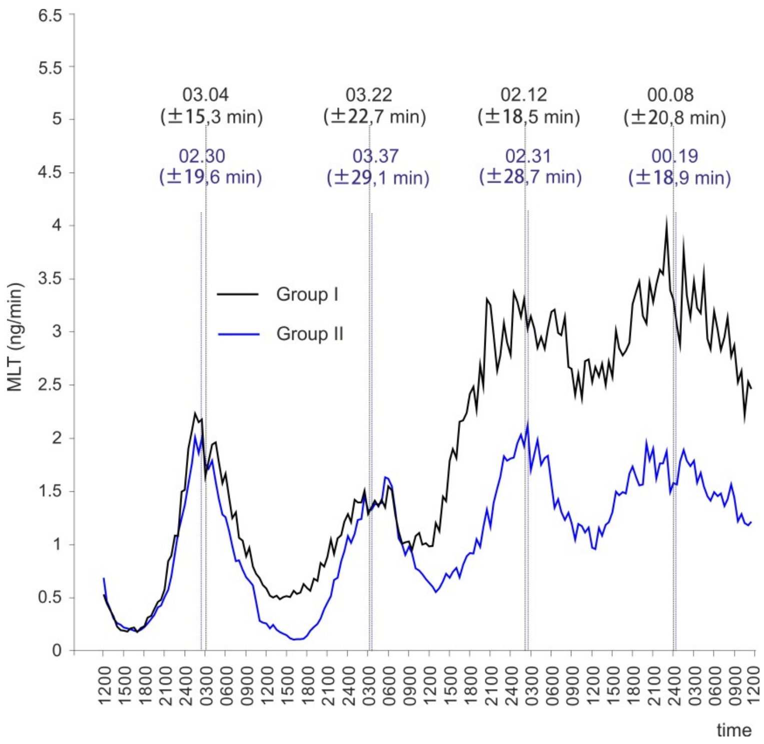

Figure S6. The phase-shifting effect of light exposure during the subjective night on circadian rhythm of MLT secretion during days III and IV (groups III and IV, experiment III). The midpoints of the peaks (clock time, mean and SEM) are marked with vertical lines. * significant (p≤0.05) shifts of the midpoints of the peaks.

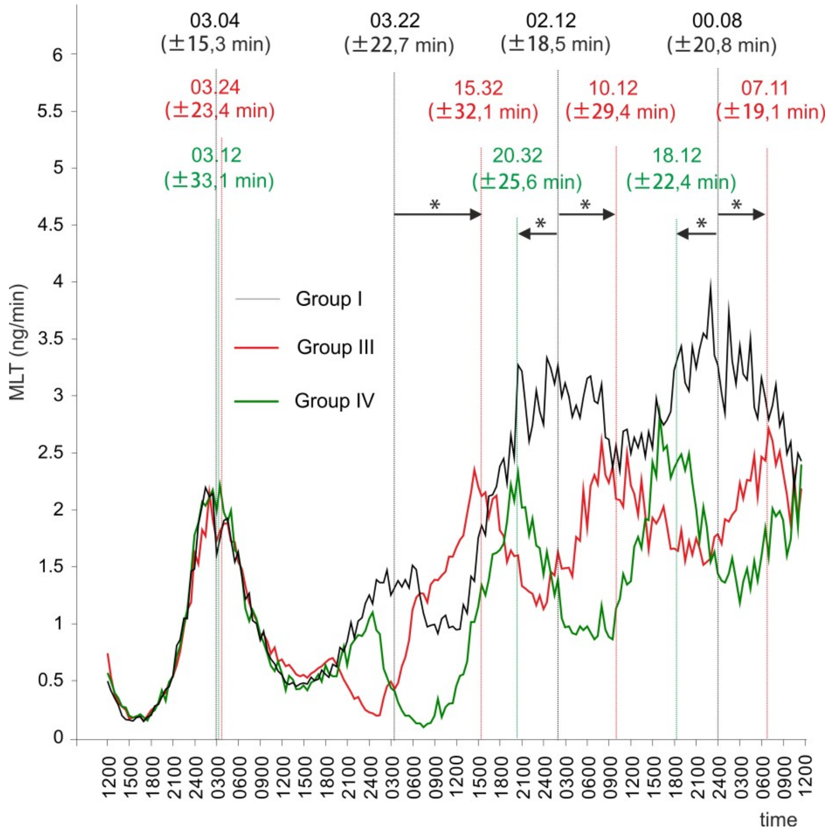

Figure S7. Stimulatory effect of darkness on MLT secretion (mean and SEM) from the pineal organs of groups II and III in experiment IV. Values labeled with different letters differ significantly (p≤0.05) between groups.

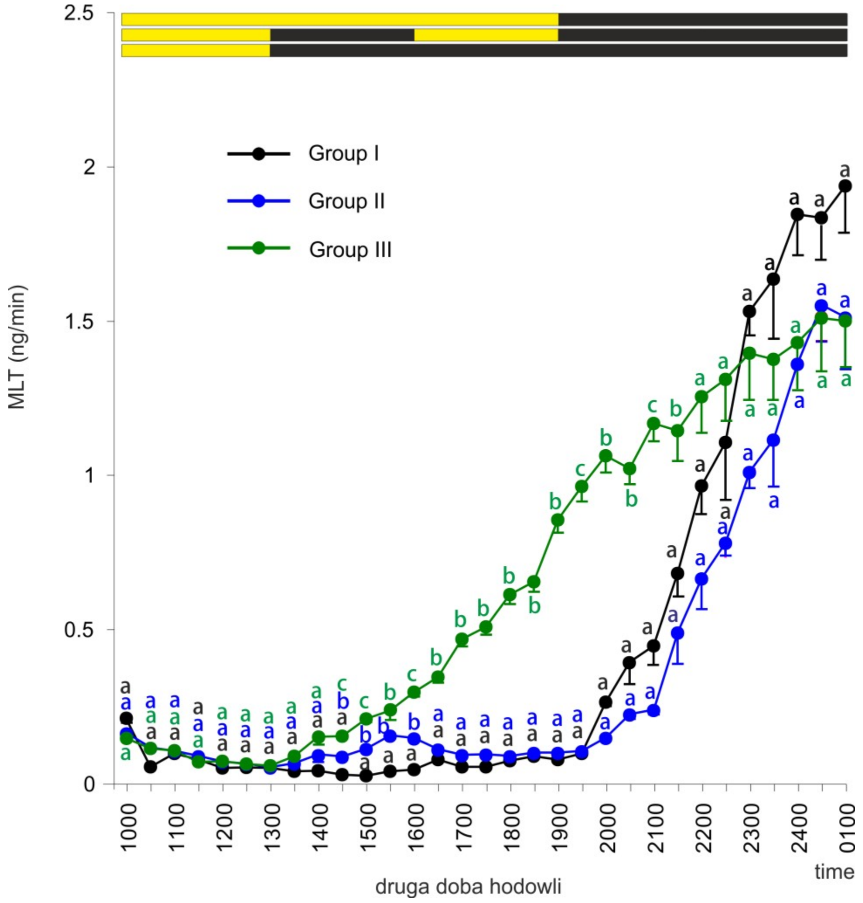

Table S1. Parameters (mean ± SEM) characterizing the rhythm of MLT secretion during day I and day III in experiment IV. The minimum and maximum points are characterized by the time clock presented in the format "hh.mm" (the standard error in minutes) and the level of secretion in ng/min. Points I50 and I75 and M are expressed in units of time clock in the format "hh.mm" (the standard error in minutes). The values labeled with different letters differ significantly (between groups) in the Duncan test (p≤0.05). n.d. - not determined.

Groups	Day I					Day III				
	Minimum		Maximum		M	Minimum		Maximum		M
	Clock time	Secretion level	Clock time	Secretion level	Clock time	Clock time	Secretion level	Clock time	Secretion level	Clock time
I	19.28 (±22.9)	0.05 (±0.01)	05.08 (±22.4)	2.07 (±0.21)	05.15 (±32.7)	09.12^{a} (±18.9)	0.41 (±0.15)	04.05^{a} (±25.3)	2.95 (±0.23)	04.27^{a} (±25.0)
II	19.15 (±17.9)	0.06 (±0.01)	05.12 (±29.8)	1.89 (±0.05)	05.08 (±19.8)	08.45^{a} (±42.2)	0.46 (±0.12)	03.37^{a} (±32.3)	3.31 (±0.28)	04.17^{a} (±31.8)
III	19.07 (±44.5)	0.06 (±0.01)	05.18 (±23.5)	2.17 (±0.07)	04.50 (±25.7)	09.30^{a} (±39.1)	0.47 (±0.24)	23.47^{b} (±41.2)	2.39 (±0.27)	00.37^{b} (±17.8)

Figure S8. Inhibitory effect of light exposure on MLT secretion (mean and SEM) from the pineal organs of groups II - IV in experiment V Values labeled with different letters differ significantly (p≤0.05) between groups.

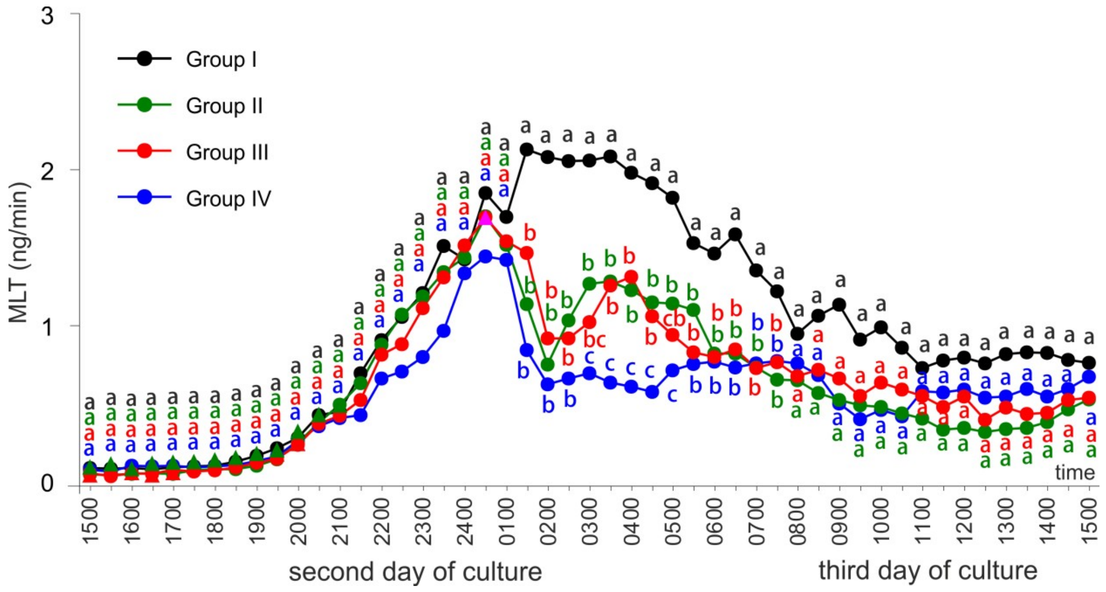

Table S2. Parameters (mean ± SEM) characterizing the rhythm of MLT secretion during day I and day III in experiment V. The minimum and maximum points are characterized by the time clock presented in the format "hh.mm" (the standard error in minutes) and the level of secretion in ng/min. Points I50 and I75 and M are expressed in units of time clock in the format "hh.mm" (the standard error in minutes). The values labeled with different letters differ significantly (between groups) in the Duncan test (p≤0.05). n.d. - not determined.

Groups	Day I					Day III				
	Minimum		Maximum		M	Minimum		Maximum		M
	Clock time	Secretion level	Clock time	Secretion level	Clock time	Clock time	Secretion level	Clock time	Secretion level	Clock time
I	19.02 (±32.7)	0.04 (±0.01)	05.38 (±32.7)	1.12 (±0.21)	05.18 (±22.9)	11.00[a] (±29.7)	0.73[a] (±0.17)	23.38[a] (±16.7)	1.95 (±0.31)	23.27[a] (±15.7)
II	19.15 (±27.7)	0.03 (±0.01)	05.32 (±19.2)	1.16 (±0.14)	05.20 (±29.8)	12.28[a] (±32.2)	0.26[b] (±0.12)	21.07[b] (±14.3)	1.39 (±0.38)	21.07[b] (±18.9)
III	19.07 (±34.7)	0.05 (±0.01)	05.31 (±32.2)	1.12 (±0.07)	05.07 (±17.4)	12.35[a] (±19.7)	0.39[b] (±0.14)	20.27[b] (±21.7)	1.71 (±0.27)	20.29[b] (±17.8)
IV	18.57 (±31.7)	0.04 (±0.01)	05.12 (±28.9)	1.17 (±0.22)	05.02 (±25.2)	9.29[b] (±20.8)	0.19[b] (±0.24)	21.02[b] (±15.7)	1.62 (±0.14)	21.34[b] (±17.4)
